# Diurnal dynamics of stress and mood during COVID-19 lockdown: a large multinational ecological momentary assessment study

**DOI:** 10.1098/rspb.2021.2480

**Published:** 2022-05-25

**Authors:** Anja C. Feneberg, Paul A. G. Forbes, Giulio Piperno, Ekaterina Pronizius, Ana Stijovic, Nadine Skoluda, Claus Lamm, Urs M. Nater, Giorgia Silani

**Affiliations:** ^1^ Department of Clinical and Health Psychology, University of Vienna, Vienna, Austria; ^2^ Department of Cognition, Emotion, and Methods in Psychology, University of Vienna, Vienna, Austria; ^3^ Research Platform The Stress of Life (SOLE)—Processes and Mechanisms underlying Everyday Life Stress, University of Vienna, Vienna, Austria

**Keywords:** COVID-19, diurnal changes, ecological momentary assessment, hair cortisol, mood, stress

## Abstract

The COVID-19 pandemic resulted in severe disruption to people's lives as governments imposed national ‘lockdowns’. Several large surveys have underlined the detrimental short- and long-term mental health consequences resulting from this disruption, but survey findings are only informative of individuals' retrospectively reported psychological states. Furthermore, knowledge on psychobiological responses to lockdown restrictions is scarce. We used smartphone-based real-time assessments in 731 participants for 7 days and investigated how individuals’ self-reported stress and mood fluctuated diurnally during lockdown in spring 2020. We found that age, gender, financial security, depressive symptoms and trait loneliness modulated the diurnal dynamics of participants' momentary stress and mood. For example, younger and less financially secure individuals showed an attenuated decline in stress as the day progressed, and similarly, more lonely individuals showed a diminished increase in calmness throughout the day. Hair collected from a subsample (*n* = 140) indicated a decrease in cortisol concentrations following lockdown, but these changes were not related to any of the assessed person-related characteristics. Our findings provide novel insights into the psychobiological impact of lockdown and have implications for how, when and which individuals might benefit most from interventions during psychologically demanding periods.

## 1. Introduction

Due to the COVID-19 pandemic, countries imposed multiple intermittent national lockdowns to slow the rate of infections. As a result, many individuals experienced significant challenges in their everyday lives, including changes in their social relationships, job status and family life. Several surveys have underlined the detrimental short- and long-term mental health consequences arising from this disruption, and identified socio-demographic and psychological characteristics that might increase the risk of negative mental health outcomes [1].

Yet, most studies linking mental health risk and resilience factors during the COVID-19 pandemic have been cross-sectional and employed retrospective measures. These approaches are prone to reporting biases and cannot accurately capture time-dependent processes [2]. To overcome these limitations, we employed an ecological momentary assessment (EMA) approach to examine *diurnal changes* in momentary stress and mood. Moreover, we determined how they are influenced by person-related characteristics under prolonged lockdown restrictions.

Typically, momentary stress tends to be higher earlier in the day and shows a decline towards the evening [3] and similar findings have been reported for energetic arousal [4]. Mood valence usually follows the opposite pattern, with lower mood in the morning and higher mood in the evening [4,5] (but see [6]). Research prior to the COVID-19 pandemic showed that depressive symptoms [7–9], age [10,11] and gender [12] modulate the diurnal dynamics of mood. Individuals with more depressive symptoms have less positive mood upon awakening [9] and show a lagged diurnal peak in mood [8]. Women, compared to men, show an earlier diurnal peak in happiness [12], and older people tend to show greater positive mood in daily life [10].

Chronic stress [13], financial status (e.g. employment [14]) and loneliness [15] are known to affect the diurnal rhythm of the hypothalamic-pituitary-adrenal (HPA) axis but it remains unknown whether these factors influence fluctuations in subjective momentary stress and mood throughout the day. That said, lonelier and more chronically stressed individuals show difficulties in emotion regulation and how they cope with stressful life events, including the COVID-19 pandemic [16,17]. Additionally, more financially insecure individuals seem to be particularly vulnerable to the effects of the pandemic [1]. Therefore, we investigated (1) potential differences in diurnal changes in momentary stress and mood in individuals with varying degrees of financial security, depressive symptoms, chronic stress and trait loneliness, and of different ages and gender during lockdown, and (2) whether concerns about the impact of COVID-19 predicted changes in momentary stress and mood over and above these person-related factors. To address these aims, we collected real-time data five times per day across 7 consecutive days while lockdown measures were in place (April–May 2020; see electronic supplementary material, S1) in 731 individuals from three different European countries.

Furthermore, we asked a subsample of participants to provide hair samples to measure changes in hair cortisol concentrations (HCC), as self-reported and endocrine measures represent different dimensions of stress [18]. Under adverse conditions, the HPA axis upregulates the release of the stress hormone cortisol [19], which is adaptive in the short term but can increase health risks in the long term [20]. HCC therefore provides a valuable insight into the psychobiological consequences of the pandemic [21]. Recent studies found that healthcare workers had higher HCC during the pandemic than in a pre-pandemic period [22,23]. Moreover, Engert *et al*. [24] found higher scores on neuroticism and extraversion predicted higher HCC during the COVID-19 pandemic. Yet the role of additional potential risk factors for HPA axis regulation following lockdown remains to be elucidated. Work prior to the pandemic has shown that gender, age and psychological variables like depression severity and loneliness modulate *salivary* cortisol responses [8,15,25,26] but it is not clear if such differences are found in hair cortisol.

In sum, we predicted that age, gender, financial security, depressive symptoms, chronic stress, trait loneliness and concerns in relation to the COVID-19 pandemic would disrupt momentary stress and mood and their fluctuations in the daily life of individuals. Furthermore, we predicted changes in HCC in response to this global stressor.

## 2. Method

### (a) Participants

To participate, individuals had to be 18 years or older, be fluent in German or Italian (as the study was performed in Austria, Germany and Italy), and own an Android device. Participants were recruited as a convenience sample [27]. Those participants who finished the study received €20 compensation and had the chance to win a €100 voucher.

The final sample consisted of 731 participants (515 women; *M*_age_ = 31.67, s.d._age_ = 11.74; table 1). The excluded participants did not differ from the included participants on any of the person-related characteristics (see electronic supplementary material, S2 for details concerning power analysis and participant attrition).

**Table 1.** Sample characteristics.

		range (sample)	range (scale)
*n*	731		
gender	515 women (70.45%)		
country of residence	Austria: *n* = 480 (65.66%)		
	Italy: *n* = 225 (30.78%)		
	Germany: *n* = 26 (3.56%)		
education	237 (32.42%) postgraduate degree		
	189 (25.85) undergraduate degree		
	270 (36.93%) high school		
	33 (4.51%) middle school		
	2 (0.27%) elementary school or none		
age (in years)	*M* = 31.67 (s.d. = 11.74)	[18.00–80.00]	
chronic stress^a^	*M* = 18.87 (s.d. = 7.34)	[1.00–40.00]	[0.00–40.00]
depressive symptoms^b^	*M* = 8.20 (s.d. = 5.19)	[0.00–27.00]	[0.00–27.00]
loneliness^c^	*M* = 38.30 (s.d. = 9.71)	[20.00–72.00]	[20.00–80.00]
financial security^d^	*M* = 58.62 (s.d. = 31.46)	[1.00–100.00]	[0.00–100.00]
COVID-19-related concerns^e^	*M* = 49.91 (s.d. = 18.49)	[2.75–99.25]	[0.00–100.00]

Note*.* The following variables were assessed only once at entry/baseline (*d*,*e*) or on the final day (*a*–*c*) with these questionnaires:

^a^Perceived Stress Scale (PSS-10) [29,30].

^b^Patient Health Questionnaire (PHQ-9) [33].

^c^The UCLA Loneliness Scale [31,32].

^d^Single item measure: ‘How financially secure do you feel at the time being?’

^e^The mean of four items measuring COVID-19-related concerns.

For hair cortisol, samples from 140 participants were available for comparing changes in HCC following lockdown restrictions. Of these, 127 participants (98 women; *M*_age_ = 34.48, s.d._age_ = 12.55; see electronic supplementary material S3 for subsample information) remained for relating HCC to person-related factors (e.g. depressive symptoms, loneliness, etc.).

### (b) Procedure

Data collection took place between 1 April and 24 April 2020 for the German-speaking sample and between 13 April and 8 May 2020 for the Italian-speaking sample. Eligible participants received an email with a personalized link to the App ‘movisensXS’ (movisens GmbH, Karlsruhe, Germany) and an electronic manual for further study instructions. Directly after downloading the app, participants filled in a brief introductory questionnaire. The EMA period started on the following day and lasted 7 consecutive days. On the following day, participants received a link to a final online survey administered through SosciSurvey (SoSci Survey GmbH, Munich, Germany), which took around 30 min to complete.

### (c) Measures

#### (i) Sociodemographic and psychometric questionnaires

During the initial questionnaire, participants entered their age, gender, country of residence and a range of other sociodemographic variables into the app (for a full list, see https://osf.io/y39qh/). Participants estimated the extent of their COVID-19-related concerns (regarding health, finances and relationships) on visual analogue scales (VAS) ranging from 0 (*not at all*) to 100 (*strongly).* As a perceived financial security measure, participants answered the question ‘How financially secure do you feel at the time being?’ on a VAS ranging from 0 (*not at all*) to 100 (*very good*). We chose a subjective measure of socio-economic status, given its stronger relationship with well-being [28].

In the final online survey, the perceived stress scale was administered to measure self-reported chronic stress during the preceding four weeks (PSS-10) [29,30]. Depressive symptoms in the prior two weeks were measured using the depressive symptom subscale of the Patient Health Questionnaire (PHQ-9) [33], and the UCLA Loneliness Scale was used to assess trait loneliness [31,32].

#### (ii) Ecological momentary assessment

The EMA protocol included five data entries per day for 7 consecutive days. Data entries were signalled semi-randomly throughout the day (between 10.00–11.00, 11.00–14.00, 14.00–17.00, 17.00–20.00). The final daily data entry was self-initiated before going to bed, with a reminder alarm at 21.00. Participants could postpone data entries for up to 30 min. Compliance was good (data were provided in 78.6% of entries). Participants took on average 2.41 min (s.d. = 1.38) for each entry (see electronic supplementary material, S4 for compliance details).

**Momentary stress**. We used a single item to measure momentary stress [34]. Participants answered the item ‘At the moment, I feel stressed’ on a VAS ranging from 0 (*not at all*) to 100 (*very much*).

**Mood dimensions**. We used an adapted version of the Multidimensional Mood Questionnaire [35] that has been validated for use in EMA studies [36]. Participants answered six bipolar items each pair of items constituted one of the three mood dimensions: mood valence (unwell–well; dissatisfied–satisfied), energetic arousal (weak–energetic; tired–awake), and calmness (tense-relaxed; restless-calm). We changed the original instrument by reverse-scoring inverted items and applying a VAS for each item ranging from 0 to 100 (higher values correspond to higher levels of the respective mood dimension).

**Additional variables**. Participants indicated what activity they were currently engaged in at the time of the data entry (working, studying or engaging in free time). We included a broad item comprising the main categories of activities that have been recommended based on previous research [37,38] in order to keep the models as parsimonious as possible. Furthermore, each assessment was time-stamped.

#### (iii) Hair cortisol

Hair collection took place between 8 May and 18 May 2020. We analysed two subsequent segments of hair per participant with a length of 1.5 cm each, reflecting approximately six weeks, respectively (for an overview of hair length in HCC studies, see [39]). The hair segment most proximal to the scalp reflected the time period from around mid-March 2020 to the end of April 2020 (i.e. the time period of the lockdown), while the subsequent hair segment reflected the six-week pre-lockdown period, ranging from around the beginning of February to mid-March 2020. For details on the cortisol extraction and analysis, see electronic supplementary material, S5.

### (d) Statistical analyses

#### (i) Momentary stress and mood

We conducted linear mixed-effects models with random intercepts and slopes using the R package lme4 [40,41], with two levels (observations on level 1 nested within individuals on level 2) and momentary stress and the three mood measures (mood valence, calmness, energetic arousal) as outcome variables, respectively.

Following state-of-the-art recommendations [42], we investigated the main effects of time of day and person-related characteristics on the respective outcome in a first model per outcome. To examine diurnal fluctuations, we included a time variable (in hours) centred on 10.00 of the respective day (EMA time). In addition, person-related factors (age, gender, COVID-19-related concerns, chronic stress, depressive symptoms, loneliness and financial security) were added simultaneously as predictors on level 2. Subsequently, for each person-related factor in each model, we included a cross-level interaction term with EMA time (e.g. EMA time × age), to investigate whether these moderated the relationship between EMA time and momentary stress and the three mood dimensions (i.e. the diurnal fluctuation). We aimed to compare the results from the models across the different outcome measures (e.g. stress versus mood valence versus calmness versus energetic arousal). Thus, to allow such a direct comparison between the dependent variables, we kept the same predictors in each model. We used Satterthwaite's method to test for significance [43]. Interactions were explored with simple slope analyses [44]. The missing data entries of participants in the final sample were excluded listwise when computing the models. Further information on missing data is reported in electronic supplementary material, S4.

All models controlled for whether participants were engaged in free time at the moment of the assessment (free time; 1 = free time, 0 = not free time). This level 1 covariate was participant-mean centred, whereas all continuous person-related factors were grand-mean centred and dummy-coded level 2 variables remained uncentred (electronic supplementary material, S6; [45]). Although we acknowledge regional differences regarding the impact of the COVID-19 pandemic on individuals residing in Austria and Italy, we did not include country of residence as a moderating factor (discussed in electronic supplementary material, S7). We kept the random effects structure ‘maximal’ [46]. Marginal and conditional *R*^2^ were used as model fit measures [47] and variance inflation was checked for all models (reported in electronic supplementary material, S8). The formulae for all models can be found in electronic supplementary material, S9.

To estimate effect sizes, we calculated semi-partial correlation coefficients (*r*) between each predictor and outcome measure [48]. *R-*values of 0.1–0.3 are interpreted as small, 0.3–0.5 as medium and greater than 0.5 as large [49].

#### (ii) Hair cortisol

Differences pre-lockdown compared to following the implementation of lockdown restrictions (post) were compared using Wilcoxon paired-samples *t*-test. As the data were strongly skewed, we log transformed the raw cortisol values and all linear regression analyses reported below were conducted using the log-transformed values. We also included pre-lockdown cortisol levels and body mass index as control variables in the model. Finally, we tested whether any of the person-related factors (age, gender, depressive symptoms, chronic stress, COVID-19-related concerns, financial security and loneliness) were associated with hair cortisol levels during the lockdown. The data and code for the hair cortisol analysis are available online (https://osf.io/fdnm7/).

## 3. Results

We report findings for each dependent variable separately. First, we report main effects (diurnal fluctuation, main effect of person-related factors). Next, we report interactions between person-related factors and EMA time to investigate how diurnal fluctuations of momentary stress and mood varied by person-related characteristics.

### (a) Descriptive results

Averaged across the whole sample, mean momentary stress was 29.97 (s.d. *=* 25.89; range [0–100]), mood valence was 63.47 (s.d. = 21.33; range [0–100]), calmness was 61.92 (s.d. = 22.35; range [0–100]) and energetic arousal was 50.97 (s.d. = 23.12; range [0–100]). For a representation of the diurnal fluctuations of momentary stress and mood dimensions see electronic supplementary material, S10, figure S3. The intraclass correlation coefficients (ICC) indicated that 46% of the variance in momentary stress mood valence and calmness was attributable to person-related differences (level 2) while 54% of the variance could be explained by momentary influences (level 1) respectively. For energetic arousal 18% of the variance was explained by person-related differences; 82% was attributable to differences at level 1. Further descriptive statistics and correlations within- and between-persons for the outcome variables and predictors are reported in electronic supplementary material S10. When comparing our sample with representative samples before and during the COVID-19 pandemic depressive symptoms and chronic stress were higher than the pre-pandemic normative values [29,50] but comparable to the values reported during the pandemic [51]. Loneliness scores, on the other hand, are comparable to those before the pandemic [32] (see electronic supplementary material, S11 for more details).

### (b) Momentary stress

Momentary stress declined throughout the day (estimate = –0.449, s.e. = 0.037, *p* < 0.001, *r* = 0.416) and engaging in free time was associated with lower stress (estimate = –7.603, s.e. = 0.639, *p* < 0.001, *r* = 0.430). Men reported higher stress levels (estimate = 1.342, s.e. = 0.615, *p* < 0.05, *r* = 0.081), as did younger participants (estimate = –0.147, s.e. = 0.047, *p* < 0.01, *r* = 0.115). Furthermore, higher chronic stress (estimate = 0.977, s.e. = 0.105, *p* < 0.001, *r* = 0.327), more severe depressive symptoms (estimate = 0.443, s.e. = 0.150, *p* < 0.01, *r* = 0.109), loneliness (estimate = 0.140 s.e. = 0.063, *p* < 0.05, *r* = 0.082) and COVID-19-related concerns (estimate = 0.073, s.e. = 0.033, *p* < 0.05, *r* = 0.082) were all significantly associated with higher momentary stress. Financial security was not a significant predictor of momentary stress (*p* = 0.236).

**Figure 1.**
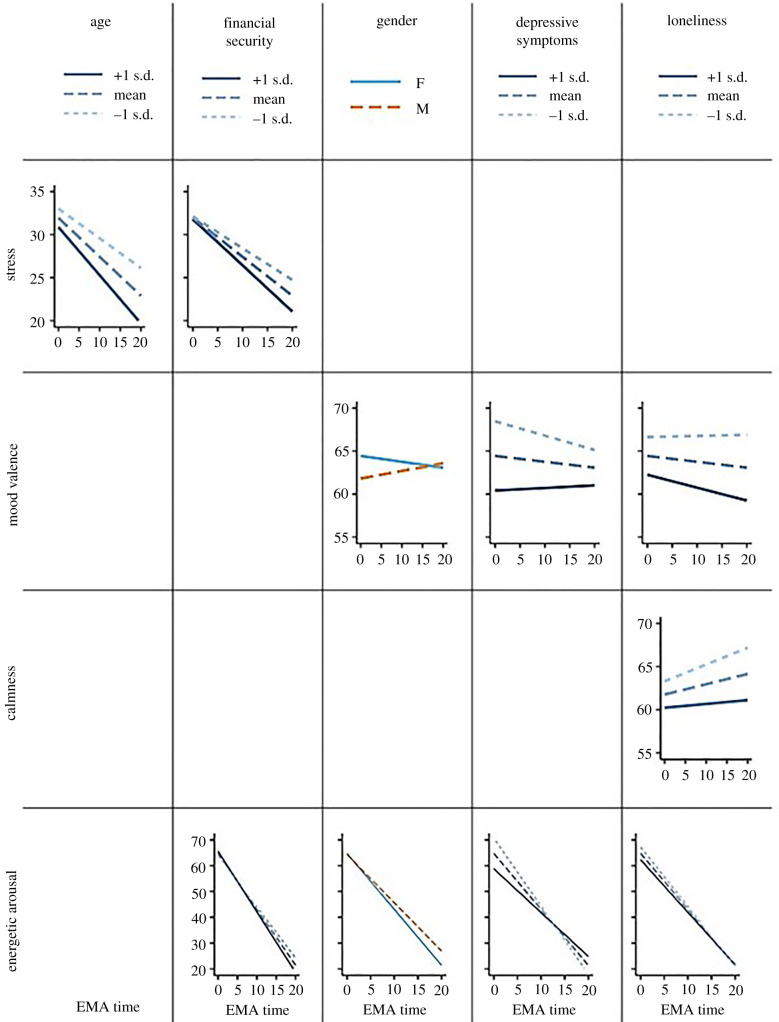
An overview of the interactions between EMA time (i.e. time passed since 10.00 in hours) and the person-related characteristics. Only significant interactions are shown. Higher values correspond to a higher level of either momentary stress, mood valence, calmness or energetic arousal. (Online version in colour.)

When taking cross-level interaction with EMA time into account (i.e. the effect of person-related factors on diurnal fluctuations), we found a significant interaction between EMA time and age (estimate = –0.009, s.e. = 0.003, *p* < 0.01, *r* = 0.110). Simple slope analysis revealed that older age (+1 s.d.) was associated with a steeper decline in momentary stress throughout the day (estimate = –0.564, s.e. = 0.052, *p* < 0.001) compared to mean-aged (estimate = –0.456, s.e. = 0.036, *p* < 0.001) and younger (–1 s.d.) participants (estimate = –0.349, s.e. = 0.050 *p* < 0.001). Furthermore, there was a significant interaction between EMA time and financial security (estimate = –0.003, s.e. = 0.001, *p* < 0.05, *r* = 0.084). As with age, at higher (+1 s.d.) levels of financial security, there was a greater decline in momentary stress throughout the day (estimate = –0.541, s.e. = 0.053, *p* < 0.001) compared to mean (estimate = –0.456, s.e. = 0.036, *p* < 0.001) or lower (–1 s.d.) levels (estimate = –0.372, s.e. = 0.052, *p* < 0.001). None of the other interactions reached significance (all *p*-values ≥ 0.092). Therefore, both younger age and less financial security were associated with a reduced decline in momentary stress throughout the day (figure 1).

### (c) Mood valence

The analysis of main effects revealed that EMA time per se was not associated with mood valence (*p* = 0.530) indicating that mood valence did not fluctuate across the day. While engaging in free time was associated with higher mood valence (estimate = 4.833, s.e. = 0.508, *p* < 0.001, *r* = 0*.*345), chronic stress (estimate = –0.725, s.e. = 0.083, *p* < 0.001, *r* = 0.308), depressive symptoms (estimate = –0.664, s.e. = 0.119, *p* < 0.001, *r* = 0.204) and loneliness (estimate = –0.227, s.e. = 0.050, *p* < 0.001, *r* = 0.201) were all associated with lower mood valence. 

When investigating cross-level interactions between person-related factors and EMA time, there was a significant yet rather small (in terms of effect size) interaction effect of gender (estimate = 0.078, s.e. = 0.034, *p* < 0.05, *r* = 0.086). The slope analysis showed no significant fluctuation of mood valence neither for women (*p* = 0.065), nor for men (*p* = 0.121).

There was also a significant interaction between EMA time and depressive symptoms (estimate = 0.019, s.e. = 0.008, *p* < 0.05, *r* = 0.086). Those participants scoring higher on depressive symptoms (+1 s.d.), while having lower mood valence, showed no significant change in mood valence throughout the day (estimate = 0.076, s.e. = 0.052, *p* = 0.147). By contrast, those with less depressive symptoms (–1 s.d.) showed a decline in mood valence throughout the day (estimate = –0.120, s.e. = 0.053, *p* < 0.05). Those with mean levels of depressive symptoms showed no significant change in mood valence throughout the day (estimate = –0.022, s.e. = 0.031, *p* = 0.477).

Furthermore, there was a significant interaction between EMA time and loneliness (estimate = –0.008, s.e. = 0.003, *p* < 0.05, *r* = 0.089). Lonelier participants (+1 s.d.) showed a decline in mood valence throughout the day (estimate = –0.103, s.e. = 0.046, *p* < 0.05), but this decline was not seen in those with mean levels of loneliness (estimate = –0.022, s.e. = 0.031, *p* = 0.478) and less lonely participants (–1 s.d.) (estimate = 0.059, s.e. = 0.046, *p* = 0.197). None of other interactions reached significance (all *p*-values ≥ 0.130).

### (d) Calmness

The analysis revealed that calmness increased throughout the day (estimate = 0.147, s.e. = 0.034, *p* < 0.001, *r* = 0.165). Not engaging in free time (estimate = 5.461, s.e. = 0.535, *p* < 0.001, *r* = 0.374), identifying as a man (estimate = –1.067, s.e. = 0.518, *p* < 0.05, *r* = 0.076), higher chronic stress (estimate = –0.931, s.e. = 0.089, *p* < 0.001, *r* =0.364), more depressive symptoms (estimate = –0.473, s.e. = 0.126, *p* < 0.001, *r* = 0.138) and greater loneliness (estimate = –0.206, s.e. = 0.053, *p* < 0.001, *r* = 0.142), were all associated with less calmness.

There was a significant interaction between EMA time and loneliness (estimate = –0.008, s.e. = 0.004, *p* < 0.05, *r* = 0.078). Less lonely participants (*–*1 s.d.) showed an increase in calmness throughout the day (estimate = 0.225, s.e. = 0.049, *p* < 0.001) as did those reporting mean levels of loneliness (estimate = 0.150, s.e. = 0.033, *p* < 0.001), whereas lonelier participants (*+*1 s.d.) did not show such an increase in calmness throughout the day (estimate = 0.075, s.e. = 0.049, *p* = 0.129) (figure 1). None of the other interactions reached significance (all *p-*values ≥ 0.152)

### (e) Energetic arousal

The analysis revealed that energetic arousal declined markedly from morning to evening (estimate = –2.063, s.e. = 0.059, *p* < 0.001, *r* = 0.793). Furthermore, not engaging in free time (estimate = 6.367, s.e. = 0.591,  *p* < 0.001, *r* = 0.392), younger age (estimate = 0.069, s.e. = 0.028,  *p* < 0.05, *r* = 0.090), identifying as a women (estimate = 1.013, s.e. = 0.369, *p* < 0.01, *r* = 0.102), higher chronic stress (estimate = –0.162, s.e. = 0.063, *p* < 0.05, *r* = 0.096), having more depressive symptoms (estimate = –0.397, s.e. = 0.090,  *p* < 0.001, *r* = 0.163) and greater loneliness (estimate = –0.142, s.e. = 0.038, *p* < 0.001, *r* = 0.137) were all associated with lower energetic arousal.

There was a significant interaction between EMA time and gender (estimate = 0.149, s.e. = 0.060, *p* < 0.05, *r* = 0.093). Women showed a steeper decline in energetic arousal throughout the day (estimate = –2.166, s.e. = 0.065, *p* < 0.001) compared to men (estimate = –1.868, s.e. = 0.100, *p* < 0.001). There was a significant interaction between EMA time and depressive symptoms (estimate = 0.091, s.e. = 0.015, *p* < 0.001, *r* = 0.227). Those participants with less severe depressive symptoms (*–*1 s.d.), while reporting greater energetic arousal overall, showed a steeper decline in energetic arousal throughout the day (estimate = –2.547, s.e. = 0.094, *p* < 0.001), compared to participants with more severe (*+*1 s.d.) depressive symptoms (estimate=−1.610, s.e. = 0.092, *p* < 0.001) or with mean levels of depressive symptoms (estimate = −2.078, s.e. = 0.055, *p* < 0.001). Similarly, there was a significant interaction between EMA time and loneliness (estimate = 0.014, s.e. = 0.006, *p* < 0.05, *r* = 0.082). Those with lower loneliness scores (*–*1 s.d.), while reporting greater energetic arousal overall, showed a steeper decline in energetic arousal throughout the day (estimate = –2.210, s.e. = 0.081, *p* < 0.001), compared to participants with higher (*+*1 s.d.) loneliness scores (estimate = –1.947, s.e. = 0.081, *p* < 0.001) and mean loneliness scores (estimate = –2.078, s.e. = 0.055, *p* < 0.001). There was also a significant interaction between EMA time and financial security (estimate = –0.006, s.e. = 0.002, *p* < 0.001, *r* = 0.118). Participants reporting greater financial security (*+*1 s.d.) showed a steeper decline in energetic arousal throughout the day (estimate = –2.262, s.e. = 0.080, *p* < 0.001) compared to participants with less (*–*1 s.d.) financial security (estimate = –1.895, s.e. = 0.079, *p* < 0.001) and mean financial security (estimate = –2.078, s.e. = 0.055, *p* < 0.001). None of the other interactions reached significance (all *p*-values ≥ 0.054).

### (f) Hair cortisol concentration

**Figure 2.**
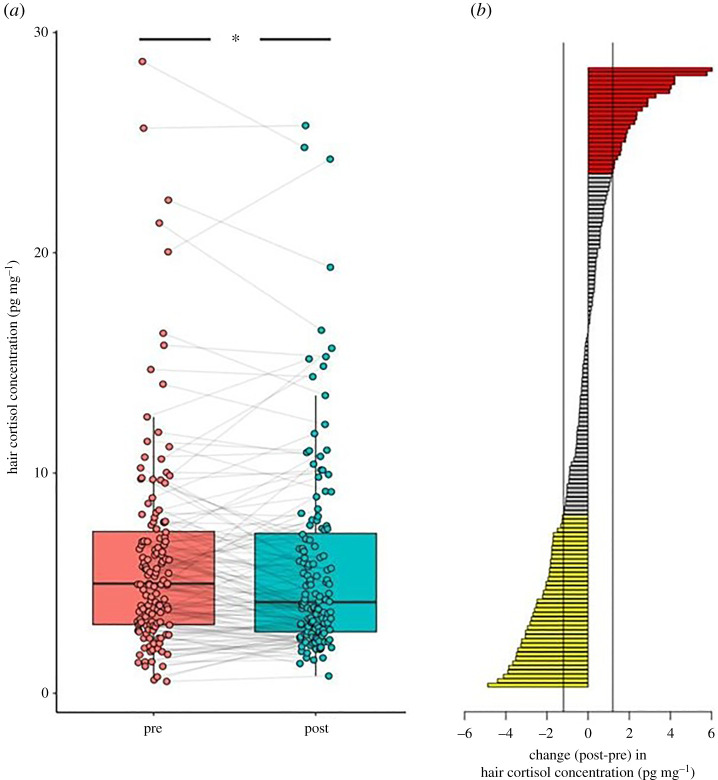
A boxplot showing hair cortisol concentration (HCC) for each subject before lockdown (pre) and after the lockdown restrictions were implemented (post). (*a*) The change in HCC after the lockdown restrictions were implemented (post) compared to before (pre) per subject (*b*). Yellow bars indicate a decrease (28% of participants), and red bars (17%) an increase, of at least 1.2 pg mg^–1^ [23]. Most participants (55%) showed no change of at least 1.2 pg mg^–1^ as shown by the grey bars. (Online version in colour.)

Overall, we found that HCC was higher before lockdown (pre-lockdown median = 4.98 pg mg^–1^, min = 0.54, 1st quartile = 3.12, 3rd quartile = 7.35, max = 28.69) compared to during lockdown (lockdown median = 4.14 pg mg^–1^, min = 0.79, 1st quartile = 2.79, 3rd quartile = 7.26, max = 25.78) and a paired Wilcoxon signed-rank test revealed that this difference was significant (*p* < 0.05; figure 2*a*).

A recent study reported 1.2 pg mg^–1^ average increase in HCC, employing a similar methodology and investigating changes as a result of the COVID-19 pandemic [23]. Compared to this criterion, in our study, 24 (17.14%) participants showed an increase in HCC of at least 1.2 pg mg^–1^ following the lockdown restrictions compared to before, whereas 39 (27.86%) participants showed a decrease of at least 1.2 pg mg^–1^, leaving 77 participants (55%) with relatively stable HCC across hair segments (figure 2*b*).

Apart from pre-lockdown HCC, which strongly predicted HCC during lockdown (estimate = 0.811, s.e. = 0.046, *t* = 17.84, *p* < 0.001), no other person-related variable was significantly associated with HCC during lockdown (all *p*-values > 0.140). When we included the difference between HCC pre- versus during lockdown as the dependent variable, again, no variables significantly predicted changes in HCC (all *p*-values > 0.290). Additional analysis revealed that changes in HCC did not predict levels of momentary stress or mood during the study period.

## 4. Discussion

We conducted an EMA study during COVID-19 lockdown to gain novel insights into the diurnal dynamics of momentary stress and mood during this psychologically taxing period. Age, gender, financial security, depressive symptoms and loneliness disrupted the diurnal dynamics of individuals' momentary stress and mood. We found a decrease in HCC following lockdown restrictions, but these were not related to any of the person-related characteristics nor to the EMA measures.

### (a) Momentary stress dynamics during lockdown

Momentary stress varied throughout the day with higher values in the morning and a decline towards the evening, supporting findings from before the COVID-19 pandemic [3]. The diurnal variation in stress was modulated by age and financial security. Younger individuals, besides displaying higher stress levels, showed a less marked decline in their momentary stress throughout the day compared to older participants. A similar effect was seen in less financially secure individuals—participants with below average financial security also showed a blunted daily decline in momentary stress. Being younger and more financially insecure could therefore act as a risk factor for increased momentary stress particularly in the evenings, which is an important time of recovery and relaxation [52].

Depressive symptoms, loneliness and chronic stress did not modulate the diurnal fluctuations of momentary stress. But individuals with more depressive symptoms, loneliness and chronic stress reported higher overall momentary stress in daily life in line with previous surveys (e.g. [53]). We found that men reported higher momentary stress than women, in contrast to other COVID-19 studies (e.g. [54]). Higher overall momentary stress levels in the daily life of men compared to women might be explained by a lower resilience [55] or by a use of less efficient coping strategies during the first lockdown period [56]. However, more research is needed to tease apart gender-related differences in terms of the effect of the COVID-19 pandemic on momentary stress levels.

### (b) Mood dynamics during lockdown

Participants reported greater calmness later in the day, whereas mood valence remained diurnally stable. A recent review found that some studies showed increases in mood valence and calmness during the day while others did not [37], and that the effects of time of day on mood tend to disappear when situational factors such as momentary activity are controlled for, which is a potential explanation for our results. Furthermore, in our sample, energetic arousal was higher in the morning and decreased during the day, and showed the biggest change throughout the day compared to the other measures, supporting previous studies [4].

Participants with more depressive symptoms and higher trait loneliness displayed lower energetic arousal, in line with prior reports [57,58] (figure 1, bottom row). Potential underlying causes may be sleep disturbances, as lower social connectedness and higher depressive symptoms have been related to poorer sleep quality during the pandemic [59]. We also found an interaction between time of the day and both depressive symptoms and loneliness, as more depressed and lonelier subjects showed an attenuated decrease in energetic arousal throughout the day. Concerning depression in particular, the same pattern of *hypoarousal* in the mornings and *hyperarousal* in the evenings has been reported previously, and could be associated with a shift in chronotype [60]. Furthermore, pre-pandemic research has consistently shown a negative relationship between loneliness and vigour [61], which could have detrimental effects on the motivation to initiate social interactions and thereby change lonely states [62].

As for loneliness and depression, we found lower financial security to be related to a less marked decline in energetic arousal throughout the day. Financial stress has been related to poorer sleep during the lockdown [63]. This suggests that lower socio-economic status could act as a risk factor for alterations in diurnal arousal, similarly to loneliness and depressive symptoms. Interestingly, financial insecurity, depression and loneliness were associated both with prolonged states of arousal *and* increased perceived stress levels in evenings. Thus, these more vulnerable individuals might benefit from stress-reducing, relaxing activities, especially in the evenings.

Alterations in the diurnal dynamics of mood valence and calmness were also identified in participants with higher depressive symptoms and loneliness. Participants who had more depressive symptoms, whilst having lower mood valence overall, tended to show a slight increase as the day progressed. This finding fits with previous work showing that individuals with more depressive symptoms tend to show a diurnal peak in positive mood later in the day [8]. It is important to note that both ours and a normative sample collected in the same period [51] suggest higher depressive symptomatology during COVID-19 lockdown compared to the pre-pandemic norm [64]. Therefore, the effects of depressive symptomatology on diurnal dynamics during this period may have been especially pronounced and may have appeared in individuals who were largely unaffected before the pandemic.

Greater trait loneliness predicted a decline in mood valence and a diminished increase in calmness throughout the day. Given that loneliness modulated diurnal dynamics of all three mood measures, lonely individuals may be a particularly vulnerable to diurnal mood disruptions. In the evenings, these participants may have become more aware of their loneliness and/or ruminated more due to the lack of distractions (e.g. work, study) present earlier in the day. Thus, interventions could be most needed towards the evening. Loneliness scores in our sample were comparable to that of a normative sample [32]. There are indeed opposing findings regarding changes in loneliness at the start of the pandemic, as it was found in some studies [65] but not in others (e.g. [66]).

Gender also modulated the diurnal dynamics of mood. Although women did not have lower mood across the study period than men, their mood valence showed a decrease during the day, while remaining stable in men. Furthermore, women reported lower energetic arousal overall, while men were less calm and showed heightened energetic arousal in the evenings. These results partially align with cross-sectional studies showing that women experienced more negative affect during lockdown [67] and further suggest gender differences in diurnal mood.

### (c) Hair cortisol before and during lockdown

Our results show a decrease of HCC following lockdown restrictions. By contrast, previous studies performed on nurses [23] and health workers [22], who were exceptionally burdened during the pandemic, reported an increase in HCC. We outline several potential explanations for this decrease. Firstly, there is evidence that individuals reported fewer ‘daily hassles’ during lockdown [68], which might be reflected in reduced cortisol output during this period. Secondly, hair segments represented the period of February/early March 2020 (pre-lockdown) and mid-March/April 2020 (lockdown), thus changes in daylight and/or temperature may have accounted for the results. Moreover, participants were constrained in their homes and were less physically active. A recent meta-analysis suggested that HCC per se is not associated with psychological variables (e.g. perceived stress, depressive symptoms) and significant correlations between HCC and self-reported psychological variables are likely to be more present in clinical and severely burdened populations [39]. Thus, it is conceivable that our sample was not burdened to a degree that affected the HPA axis in a consistent manner across participants.

### (d) Limitations and implications

This study lacks a ‘baseline’ assessment, which would have allowed us to establish the extent to which our results are specific to lockdown. Our sampling regime also started at 10.00 and might have missed potential early morning changes in perceived momentary stress and mood [69].

Additionally, we included all person-related factors simultaneously in our models so could not identify potential combinations of person-related factors that influence the diurnal dynamics of momentary stress and mood. One strength of this approach, however, is that it allowed us to determine each person-related factor's unique contribution to the diurnal dynamics of stress and mood while controlling for all other factors.

On a broader level, our findings have important theoretical implications for approaches to mental health. The results show the importance of considering not only risk and resilience *trait* factors (e.g. chronic stress, loneliness) but also momentary *states* (e.g. disrupted diurnal mood fluctuations). While trait measures tend to aggregate across several days or weeks, EMA approaches can reveal moment-to-moment changes in psychological states. This could provide not only a more fine-grained understanding of how individuals respond to treatments but could also help to anticipate periods of acute distress enabling prompt intervention [70].

## 5. Conclusion and future directions

The present study emphasizes the need to consider the diurnal dynamics of mental health indicators during psychologically taxing periods. The fact that person-related characteristics, such as younger age, loneliness and depressive symptoms, were associated with changes in diurnal fluctuations raises a question about underlying psychobiological and behavioural mechanisms (e.g. sleep patterns, maladaptive coping behaviours) and, additionally, hints towards specific time windows for the implementation of interventions. Although HCC decreased following lockdown restrictions, the person-related factors were unrelated to HCC. Finally, treatments aiming to improve mental health in individuals under lockdown restrictions might be particularly effective when delivered contingent on individuals' momentary needs. In this regard, investigations into the interplay of individual characteristics, daily momentary stress/mood dynamics and time point of treatment delivery seems an appealing avenue for future research in the context of precision medicine.

## Data Availability

An overview of all items and questionnaires employed in the context of the larger project are available through https://osf.io/rzqn6/. The associated data and code for all models are available online (https://osf.io/fdnm7/). Electronic supplementary material is available online [71].

## References

[1] Xiong J et al. 2020 Impact of COVID-19 pandemic on mental health in the general population: a systematic review. J. Affect. Disord. **277**, 55-64. (10.1016/j.jad.2020.08.001) 32799105 PMC7413844

[2] Shiffman S, Stone AA, Hufford MR. 2008 Ecological momentary assessment. Annu. Rev. Clin. Psychol. **4**, 1-32. (10.1146/annurev.clinpsy.3.022806.091415) 18509902

[3] Zawadzki MJ et al. 2019 Understanding stress reports in daily life: a coordinated analysis of factors associated with the frequency of reporting stress. J. Behav. Med. **42**, 545-560. (10.1007/s10865-018-00008-x) 30600403 PMC6526071

[4] Wood C, Magnello M. 1992 Diurnal changes in perceptions of energy and mood. J. R Soc. Med. **85**, 191. 1433057 10.1177/014107689208500404 PMC1294720

[5] Stone AA, Smyth JM, Pickering T, Schwartz J. 1996 Daily mood variability: form of diurnal patterns and determinants of diurnal patterns. J. Appl. Soc. Psychol. **26**, 1286-1305. (10.1111/j.1559-1816.1996.tb01781.x)

[6] Golder SA, Macy MW. 2011 Diurnal and seasonal mood vary with work, sleep, and daylength across diverse cultures. Science **333**, 1878-1881. (10.1126/science.1202775) 21960633

[7] Cowdry. 1991 Mood variability: a study of four groups. Am. J. Psychiatry **148**, 1505-1511. (10.1176/ajp.148.11.1505) 1928464

[8] Peeters F, Berkhof J, Delespaul P, Rottenberg J, Nicolson NA. 2006 Diurnal mood variation in major depressive disorder. Emotion **6**, 383-391. (10.1037/1528-3542.6.3.383) 16938080

[9] Murray G. 2007 Diurnal mood variation in depression: a signal of disturbed circadian function? J. Affect. Disord. **102**, 47-53. (10.1016/j.jad.2006.12.001) 17239958

[10] Carstensen LL, Turan B, Scheibe S, Ram N, Ersner-Hershfield H, Samanez-Larkin GR, Brooks KP, Nesselroade JR. 2011 Emotional experience improves with age: evidence based on over 10 years of experience sampling. Psychol. Aging **26**, 21-33. (10.1037/a0021285) 20973600 PMC3332527

[11] Scheibe S, English T, Tsai JL, Carstensen LL. 2013 Striving to feel good: ideal affect, actual affect, and their correspondence across adulthood. Psychol. Aging **28**, 160-171. (10.1037/a0030561) 23106153 PMC3756228

[12] Adan A, Sánchez-Turet M. 2001 Gender differences in diurnal variations of subjective activation and mood. Chronobiol. Int. **18**, 491-502. (10.1081/CBI-100103971) 11475418

[13] Starr LR, Dienes K, Li YI, Shaw ZA. 2019 Chronic stress exposure, diurnal cortisol slope, and implications for mood and fatigue: moderation by multilocus HPA-axis genetic variation. Psychoneuroendocrinology **100**, 156-163. (10.1016/j.psyneuen.2018.10.003) 30340064

[14] Ockenfels MC, Porter L, Smyth J, Kirschbaum C, Hellhammer DH, Stone AA. 1995 Effect of chronic stress associated with unemployment on salivary cortisol: overall cortisol levels, diurnal rhythm, and acute stress reactivity. Psychosom. Med. **57**, 460-467. (10.1097/00006842-199509000-00008) 8552737

[15] Doane LD, Adam EK. 2010 Loneliness and cortisol: momentary, day-to-day, and trait associations. Psychoneuroendocrinology **35**, 430-441. (10.1016/j.psyneuen.2009.08.005) 19744794 PMC2841363

[16] Gamonal-Limcaoco S, Montero-Mateos E, Lozano-López MT, Maciá-Casas A, Matías-Fernández J, Roncero C. 2021 Perceived stress in different countries at the beginning of the coronavirus pandemic. Int. J. Psychiatry Med. **0**, 1-14. (10.1177/00912174211033710) PMC9209880 34266339

[17] Rumas R, Shamblaw AL, Jagtap S, Best MW. 2021 Predictors and consequences of loneliness during the COVID-19 pandemic. Psychiatry Res. **300**, 113934. (10.1016/j.psychres.2021.113934) 33882398 PMC9755111

[18] Nater UM. 2018 The multidimensionality of stress and its assessment. Brain Behav. Immun. **73**, 159-160. (10.1016/j.bbi.2018.07.018) 30041012

[19] Chrousos GP. 2009 Stress and disorders of the stress system. Nat. Rev. Endocrinol. **5**, 374-381. (10.1038/nrendo.2009.106) 19488073

[20] McEwen BS. 1998 Stress, adaptation, and disease: allostasis and allostatic load. Ann. N Y Acad. Sci. **840**, 33-44. (10.1111/j.1749-6632.1998.tb09546.x) 9629234

[21] Stalder T, Kirschbaum C. 2012 Analysis of cortisol in hair—state of the art and future directions. Brain Behav. Immun. **26**, 1019-1029. (10.1016/j.bbi.2012.02.002) 22366690

[22] Ibar C et al. 2021 Evaluation of stress, burnout and hair cortisol levels in health workers at a University Hospital during COVID-19 pandemic. Psychoneuroendocrinology **128**, 105213. (10.1016/j.psyneuen.2021.105213) 33845387 PMC8015376

[23] Rajcani J, Vytykacova S, Solarikova P, Brezina I. 2021 Stress and hair cortisol concentrations in nurses during the first wave of the COVID-19 pandemic. Psychoneuroendocrinology **129**, 105245. (10.1016/j.psyneuen.2021.105245) 33951563 PMC8078045

[24] Engert V, Blasberg JU, Köhne S, Strauss B, Rosendahl J. 2021 Resilience and personality as predictors of the biological stress load during the first wave of the COVID-19 pandemic in Germany. Transl. Psychiatry **11**, 443. (10.1038/s41398-021-01569-3) 34455419 PMC8401367

[25] Dedovic K, Ngiam J. 2015 The cortisol awakening response and major depression: examining the evidence. Neuropsychiatr Dis. Treat. **11**, 1181-1189. 25999722 10.2147/NDT.S62289 PMC4437603

[26] Strahler J, Skoluda N, Kappert MB, Nater UM. 2017 Simultaneous measurement of salivary cortisol and alpha-amylase: application and recommendations. Neurosci. Biobehav. Rev. **83**, 657-677. (10.1016/j.neubiorev.2017.08.015) 28864234

[27] Bock O, Baetge I, Nicklisch A. 2014 hroot: Hamburg registration and organization online tool. Eur. Econ. Rev. **71**, 117-120. (10.1016/j.euroecorev.2014.07.003)

[28] Adler NE, Epel ES, Castellazzo G, Ickovics JR. 2000 Relationship of subjective and objective social status with psychological and physiological functioning: preliminary data in healthy, White women. Health Psychol. **19**, 586. (10.1037/0278-6133.19.6.586) 11129362

[29] Klein EM, Brähler E, Dreier M, Reinecke L, Müller KW, Schmutzer G, Wolfling K, Beutel ME. 2016 The German version of the perceived stress scale–psychometric characteristics in a representative German community sample. BMC Psychiatry **16**, 1-10. (10.1186/s12888-016-0875-9) 27216151 PMC4877813

[30] Mondo M, Sechi C, Cabras C. 2021 Psychometric evaluation of three versions of the Italian perceived stress scale. Curr. Psychol. **40**, 1884-1892. (10.1007/s12144-019-0132-8)

[31] Elbing E. 1991 Einsamkeit: Psychologische Konzepte, Forschungsbefunde und Treatmentansätze. Göttingen, Germany: Hogrefe.

[32] Russell DW. 1996 UCLA Loneliness Scale (version 3): reliability, validity, and factor structure. J. Pers. Assess. **66**, 20-40. (10.1207/s15327752jpa6601_2) 8576833

[33] Löwe B, Spitzer R, Zipfel S, Herzog W. 2002 Gesundheitsfragebogen für patienten (PHQ-D). Komplettversion Kurzform Testmappe Mit Man Fragebögen Schablonen **2**, 90-93.

[34] Lesage FX, Berjot S, Deschamps F. 2012 Clinical stress assessment using a visual analogue scale. Occup. Med. **62**, 600-605. (10.1093/occmed/kqs140) 22965867

[35] Steyer R, Schwenkmezger P, Notz P, Eid M. 1997 Der Mehrdimensionale Befindlichkeitsfragebogen (MDBF). Handanweisung. Göttingen, Germany: Hogrefe.

[36] Wilhelm P, Schoebi D. 2007 Assessing mood in daily life. Eur. J. Psychol. Assess **23**, 258-267. (10.1027/1015-5759.23.4.258)

[37] de Vries LP, Baselmans BML, Bartels M. 2020 Smartphone-based ecological momentary assessment of well-being: a systematic review and recommendations for future studies. J. Happiness Stud. **22**, 2361-2408. (10.1007/s10902-020-00324-7) 34720691 PMC8550316

[38] Fahrenberg J, Leonhart R, Foerster F. 2002 Alltagsnahe Psychologie mit hand-held PC und physiologischem Mess-System. Bern, Switzerland: Hans Huber.

[39] Stalder T, Steudte-Schmiedgen S, Alexander N, Klucken T, Vater A, Wichmann S, Kirschbaum C, Miller R. 2017 Stress-related and basic determinants of hair cortisol in humans: a meta-analysis. Psychoneuroendocrinology **77**, 261-274. (10.1016/j.psyneuen.2016.12.017) 28135674

[40] Bates D, Mächler M, Bolker B, Walker S. 2014 Fitting linear mixed-effects models using lme4. See http://arxiv.org/abs/1406.5823.

[41] R Core Team. 2020 *R: a language and environment for statistical computing*. Vienna, Austria: R Foundation for Statistical Computing.

[42] Aguinis H, Gottfredson RK, Culpepper SA. 2013 Best-practice recommendations for estimating cross-level interaction effects using multilevel modeling. J. Manage. **39**, 1490-1528. (10.1177/0149206313478188)

[43] Kuznetsova A, Brockhoff PB, Christensen RHB. 2017 lmerTest package: tests in linear mixed effects models. J. Stat. Softw. **82**, 1-26. (10.18637/jss.v082.i13)

[44] Long JA, Long MJA. 2019 Package ‘interactions’. See https://interactions.jacob-long.com.

[45] Enders CK, Tofighi D. 2007 Centering predictor variables in cross-sectional multilevel models: a new look at an old issue. Psychol. Methods **12**, 121. (10.1037/1082-989X.12.2.121) 17563168

[46] Barr DJ, Levy R, Scheepers C, Tily HJ. 2013 Random effects structure for confirmatory hypothesis testing: keep it maximal. J. Mem. Lang. **68**, 255-278. (10.1016/j.jml.2012.11.001) PMC3881361 24403724

[47] Nakagawa S, Schielzeth H. 2013 A general and simple method for obtaining R2 from generalized linear mixed-effects models. Methods Ecol. Evol. **4**, 133-142. (10.1111/j.2041-210x.2012.00261.x)

[48] Jaeger BC, Edwards LJ, Das K, Sen PK. 2017 An R2 statistic for fixed effects in the generalized linear mixed model. J. Appl. Stat. **44**, 1086-1105. (10.1080/02664763.2016.1193725)

[49] Cohen J. 1992 A power primer. Psychol. Bull. **112**, 155. (10.1037/0033-2909.112.1.155) 19565683

[50] Kocalevent RD, Hinz A, Brahler E. 2013 Standardization of the depression screener Patient Health Questionnaire (PHQ-9) in the general population. Gen. Hosp. Psychiatry **35**, 551-555. (10.1016/j.genhosppsych.2013.04.006) 23664569

[51] Pieh C, Budimir S, Probst T. 2020 The effect of age, gender, income, work, and physical activity on mental health during coronavirus disease (COVID-19) lockdown in Austria. J. Psychosom. Res. **136**, 110186. (10.1016/j.jpsychores.2020.110186) 32682159 PMC7832650

[52] Sonnentag S, Binnewies C, Mojza EJ. 2008 Did you have a nice evening?’ A day-level study on recovery experiences, sleep, and affect. J. Appl. Psychol. **93**, 674-684. (10.1037/0021-9010.93.3.674) 18457495

[53] Probst T, Budimir S, Pieh C. 2020 Depression in and after COVID-19 lockdown in Austria and the role of stress and loneliness in lockdown: a longitudinal study. J. Affect. Disord. **277**, 962-963. (10.1016/j.jad.2020.09.047) 33065839 PMC7487145

[54] Gómez-Salgado J, Andres-Villas M, Dominguez-Salas S, Diaz-Milanes D, Ruiz-Frutos C. 2020 Related health factors of psychological distress during the COVID-19 pandemic in Spain. Int. J. Environ. Res. Public Health **17**, 3947. (10.3390/ijerph17113947) 32498401 PMC7312369

[55] Sánchez-Teruel D, Robles-Bello MA, Valencia-Naranjo N. 2021 Do psychological strengths protect college students confined by COVID-19 to emotional distress? The role of gender. Pers. Individ Differ. **171**, 110507. (10.1016/j.paid.2020.110507) PMC9045810 35502314

[56] Prowse R, Sherratt F, Abizaid A, Gabrys RL, Hellemans KG, Patterson ZR, Mcquaid RJ. 2021 Coping with the COVID-19 pandemic: examining gender differences in stress and mental health among university students. Front. Psychiatry **12**, 439. (10.3389/fpsyt.2021.650759) PMC8058407 33897499

[57] Vaccarino AL, Sills TL, Evans KR, Kalali AH. 2008 Prevalence and association of somatic symptoms in patients with major depressive disorder. J. Affect. Disord. **110**, 270-276. (10.1016/j.jad.2008.01.009) 18280580

[58] Hawkley LC, Cacioppo JT. 2010 Loneliness matters: a theoretical and empirical review of consequences and mechanisms. Ann. Behav. Med. **40**, 218-227. (10.1007/s12160-010-9210-8) 20652462 PMC3874845

[59] Ernstsen L, Havnen A. 2021 Mental health and sleep disturbances in physically active adults during the COVID-19 lockdown in Norway: does change in physical activity level matter? Sleep Med. **77**, 309-312. (10.1016/j.sleep.2020.08.030) 32951994

[60] Antypa N, Vogelzangs N, Meesters Y, Schoevers R, Penninx BW. 2016 Chronotype associations with depression and anxiety disorders in a large cohort study. Depress Anxiety **33**, 75-83. (10.1002/da.22422) 26367018

[61] Hawkley LC, Preacher KJ, Cacioppo JT. 2010 Loneliness impairs daytime functioning but not sleep duration. Health Psychol. Off. J. Div. Health Psychol. Am. Psychol. Assoc. **29**, 124-129. (10.1037/a0018646) PMC2841303 20230084

[62] Holding BC, Sundelin T, Schiller H, Akerstedt T, Kecklund G, Axelsson J. 2020 Sleepiness, sleep duration, and human social activity: an investigation into bidirectionality using longitudinal time-use data. Proc. Natl Acad. Sci. USA **117**, 21 209-21 217. (10.1073/pnas.2004535117) 32817530 PMC7474602

[63] Robillard R et al. 2020 Social, financial and psychological stress during an emerging pandemic: observations from a population survey in the acute phase of COVID-19. BMJ Open **10**, e043805. (10.1136/bmjopen-2020-043805) PMC7735085 33310814

[64] Kroenke K, Spitzer RL, Williams JBW. 2001 The PHQ-9. J. Gen. Intern. Med. **16**, 606-613. (10.1046/j.1525-1497.2001.016009606.x) 11556941 PMC1495268

[65] Mayerl H, Stolz E, Freidl W. 2021 Longitudinal effects of COVID-19-related loneliness on symptoms of mental distress among older adults in Austria. Public Health **200**, 56-58. (10.1016/j.puhe.2021.09.009) 34678551 PMC8479381

[66] Buecker S, Horstmann KT, Krasko J, Kritzler S, Terwiel S, Kaiser T, Luhmann M. 2020 Changes in daily loneliness for German residents during the first four weeks of the COVID-19 pandemic. Soc. Sci. Med. **265**, 113541. (10.1016/j.socscimed.2020.113541) 33248868

[67] Talevi D, Socci V, Carai M, Carnaghi G, Faleri S, Trebbi E, di Bernardo A, Capelli F, Pacitti F. 2020 Mental health outcomes of the COVID-19 pandemic. Riv Psichiatr. **55**, 137-144. (10.1708/3382.33569) 32489190

[68] Ahrens KF et al. 2021 Impact of COVID-19 lockdown on mental health in Germany: longitudinal observation of different mental health trajectories and protective factors. Transl Psychiatry **11**, 1-10. (10.1038/s41398-021-01508-2) 34282129 PMC8287278

[69] Scott SB, Sliwinski MJ, Blanchard-Fields F. 2013 Age differences in emotional responses to daily stress: the role of timing, severity, and global perceived stress. Psychol. Aging **28**, 1076-1087. (10.1037/a0034000) 24364410 PMC3874135

[70] Husen K, Rafaeli E, Rubel JA, Bar-Kalifa E, Lutz W. 2016 Daily affect dynamics predict early response in CBT: feasibility and predictive validity of EMA for outpatient psychotherapy. J. Affect. Disord. **206**, 305-314. (10.1016/j.jad.2016.08.025) 27662571

[71] Feneberg AC, Forbes PAG, Piperno G, Pronizius E, Stijovic A, Skoluda N, Nater UM, Silani G. 2022 Diurnal dynamics of stress and mood during COVID-19 lockdown: a large multinational ecological momentary assessment study. Figshare. (10.6084/m9.figshare.c.5964970) PMC9130787 35611528

